# Author response to: Global surgery education in Europe: a landscape analysis

**DOI:** 10.1093/bjsopen/zrac038

**Published:** 2022-03-08

**Authors:** Lotta Velin, Adriana C. Panayi, Iris Lebbe, Emmanuelle Koehl, Gauthier Willemse, Dominique Vervoort

**Affiliations:** Centre for Teaching & Research in Disaster Medicine and Traumatology (KMC), Department of Biomedical and Clinical Sciences, Linköping University, Linköping, Sweden; Division of Plastic Surgery, Department of Surgery, Brigham and Women’s Hospital, Harvard Medical School, Boston, MA, USA; School of Clinical Medicine, University of Cambridge, Cambridge, UK; Faculty of Medicine, Katholieke Universiteit Leuven, Leuven, Belgium; Harvard T.H. Chan School of Public Health, Boston, MA, USA; Harvard T.H. Chan School of Public Health, Boston, MA, USA; Faculty of Medicine, Katholieke Universiteit Leuven, Leuven, Belgium; Institute of Health Policy, Management and Evaluation, University of Toronto, Toronto, ON, Canada

*Dear Editor*

We read the comment from Dr Bandyopadhyay, questioning our findings that show only 16 global surgery centres in Europe, with great interest^1^. We thank the author for providing us with an opportunity to expand on the limitations of our analysis and acknowledge that more institutions, such as those presented by the author, are involved in surgical care delivery in variable-resource contexts. Our analysis focused exclusively on academic global surgery initiatives and educational programmes directly associated with medical schools. Although non-governmental organizations such as KidsOR (founded in Scotland) and the Global Surgery Foundation (located in Switzerland), and student initiatives such as InciSioN chapters, were not captured by our analysis, they are central to the European global surgery landscape. Similarly, although not hosting traditional institution-based learning, subregional initiatives such as the Nordic Network for Global Surgery and Anesthesia in the Nordic countries and the German Society for Global Surgery in Germany facilitate research and educational collaborations between institutions and individuals.

We excluded non-academic initiatives for multiple reasons. First, many informal ad hoc opportunities, such as student chapters or projects based on personal partnerships, may not be described online, and were excluded to ensure consistency. Second, most hospitals have some individuals working clinically abroad (e.g. annual ‘missions’) with some hospitals even having long-term relationships established. These initiatives, however, are rarely established at the university level, as reflected in our analysis. Third, trainees often have limited opportunities to meaningfully participate in clinical initiatives abroad which are not based on institutional collaborations. Many, albeit certainly not all, of these fly-in ‘missions’ are marked by power asymmetries contradicting the spirit of equity emphasized in the definition of global surgery. Overall, our focus on academic initiatives sought to serve as a proxy for visibility and accessibility for trainees to start to engage as future global surgery leaders.
